# CYP genes in osteosarcoma: Their role in tumorigenesis, pulmonary metastatic microenvironment and treatment response

**DOI:** 10.18632/oncotarget.15869

**Published:** 2017-03-03

**Authors:** Alini Trujillo-Paolillo, Francine Tesser-Gamba, Antonio Sergio Petrilli, Maria Teresa de Seixas Alves, Reynaldo Jesus Garcia Filho, Renato de Oliveira, Silvia Regina Caminada de Toledo

**Affiliations:** ^1^ Genetics Laboratory, Pediatric Oncology Institute (IOP/GRAACC), Federal University of Sao Paulo, Rua Botucatu, Vila Clementino, Sao Paulo SP, 04023-062, Brazil; ^2^ Department of Clinical and Experimental Oncology, Federal University of Sao Paulo, Rua Dr. Diogo de Faria, Vila Clementino, Sao Paulo SP, 04037-003, Brazil; ^3^ Pediatric Oncology Institute (IOP/GRAACC), Department of Pediatrics, Federal University of Sao Paulo, Rua Botucatu, Vila Clementino, Sao Paulo SP, 04023-062, Brazil; ^4^ Department of Pathology, Federal University of Sao Paulo, Rua Botucatu, Vila Clementino, Sao Paulo SP, 04023-062, Brazil; ^5^ Department of Orthopedic Surgery and Traumatology, Federal University of Sao Paulo, Rua Borges Lagoa, Vila Clementino, Sao Paulo SP, 04038-031, Brazil; ^6^ Department of Thoracic Surgery, Federal University of Sao Paulo, Rua Napoleao de Barros, Vila Clementino SP, 04024-002, Brazil; ^7^ Department of Morphology and Genetics, Federal University of Sao Paulo, Rua Botucatu, Vila Clementino, Sao Paulo SP, 04023-062, Brazil

**Keywords:** osteosarcoma, cytochrome P-450, osteosarcoma cell line, tumor microenvironment, treatment response

## Abstract

Osteosarcoma (OS) is the most common malignant bone tumor in children and adolescents. The present study investigated the expression of Cytochrome P-450 (*CYP*) genes: *CYP1A2*, *CYP3A4* and *CYP3A5* by qRT-PCR in 135 specimens obtained from OS patients, including biopsy (pre-chemotherapy), tumor resected in surgery (post-chemotherapy), adjacent bone to tumor (nonmalignant tissue), pulmonary metastasis and adjacent lung to metastasis (nonmalignant tissue). Normal bone and normal lung tissues were used as control. We also investigated in five OS cell lines the modulation of *CYPs* expression by cisplatin, doxorubicin and methotrexate. As result, the adjacent lung specimens presented *CYP1A2* overexpression compared to the normal lung (p=0.0256). Biopsy specimens presented lower *CYP3A4* expression than normal bone (p=0.0314). The overexpression of both *CYP1A2* and *CYP3A4* in post-chemotherapy specimens were correlated with better event free-survival (p=0.0244) and good response (p=0.0484), respectively. Furthermore, *in vitro* assays revealed that *CYP1A2* was upregulated by doxorubicin (p=0.0034); *CYP3A4* was upregulated by cisplatin, doxorubicin and methotrexate (p=0.0004, p=0.0024, p<0.0001, respectively); and *CYP3A5* was downregulated by doxorubicin (p=0.0285) and upregulated in time-dependent manner by methotrexate (p=0.0239). In conclusion, our findings suggest that *CYP* genes play an important role in OS tumorigenesis, at primary and metastatic sites, as well in treatment response.

## INTRODUCTION

Osteosarcoma (OS) originates from primitive mesenchymal cells and it is the most common malignant bone tumor in children and adolescents [1]. OS treatment still uses the same drugs since 1980s and survival rates have not been improved since then, consequently translational research is required to identify targets for novel treatment modalities [2]. Cytochrome P-450 (CYP) enzymes participate in the phase I of drug metabolism, mediating drug oxidation, reduction and hydrolysis reactions, therefore activating or deactivating drugs during these processes [3, 4]. The CYP enzymes also mediate metabolic activation of procarcinogens, thus they could play a role in both tumorigenesis and treatment response [4].

Although many CYP members detoxify anticancer drugs used in OS treatment, such as ifosfamide, doxorubicin, etoposide and cisplatin [5, 6], the impact of CYP to treatment response and prognosis in OS needs to be further explored and defined [5]. Dhaini et al. found a higher frequency of CYP1A2 expression detection in OS biopsy fragments than in most cancers investigated (80%) [7]. *CYP1A2* gene expression has already been detected in human lung tissue [8], the main metastatic site in OS. In OS patients, the variant rs4646437 in *CYP3A4* gene has been associated with 5-year progression-free survival [9]. Mensah-Osman et al. verified that Pregnane Xenobiotic Receptor (PXR) plays a critical role in *CYP3A4* gene expression regulation in OS and its activation may influence the chemotherapy effect in target genes implicating drug resistance [10]. In addition, analyses of CYP3A4/5 expression in OS biopsies revealed that high expression might predict metastasis and poor prognosis in OS [7].

The CYP enzymes are involved in the biosynthesis or catabolism of steroid hormones, bile acids, fat-soluble vitamins, fatty acids, and eicosanoids, which play a role in important pathways [11]. The CYP1A2 enzyme can convert several procarcinogens, including polycyclic aromatic hydrocarbons (PAHs), heterocyclic amines, and aflatoxin B1 (AFB1) to reactive electrophiles that can interact with DNA and proteins [12]. Thus, differences in CYP1A2 activity may influence individual susceptibility to cancer risk [4]. Polymorphisms in the *CYP1A2* gene have already been related with cancer risk in liver, lung, stomach, pancreas, breast, endometrium and ovarian [13–19]. As for drugs, CYP3A enzyme also plays an important role in the metabolism of several endogenous substances, including testosterone and progesterone [20]. Polymorphism in *CYP3A4* gene was associated with early puberty [21] and has been associated with a high grade and advanced stage of prostate cancers [22]. Furthermore, a meta-analysis showed that polymorphism in *CYP3A5* gene may increase the cancer risk, especially in acute leukemia, chronic leukemia, and colorectal cancer [23]. The major sources of variability in CYP activities are environmental influences, including inhibition or induction by drugs; biological factors including sex and physiological determinants, such as hormonal status, disease, and circadian rhythms; and genetic polymorphisms in both *CYP* genes and their regulators [20].

Therefore, the aim of the present study was to investigate if the expression levels of *CYP1A2*, *CYP3A4* and *CYP3A5* genes play a role in OS tumorigenesis and treatment response. Thus, we analyzed different specimens in time for each OS patient during the treatment and disease progression. As well, we investigated nonmalignant tissues surrounding the tumor, such as bone and lung tissues that could play an important role in priming the microenvironment for OS establishment. This study also investigated if the major drugs used in OS treatment and their different combinations could modulate *CYP* genes expression in OS cell lines.

## RESULTS

### Gene expression of *CYP1A2*, *CYP3A4* and *CYP3A5* in OS patients and controls

**Figure 1:**
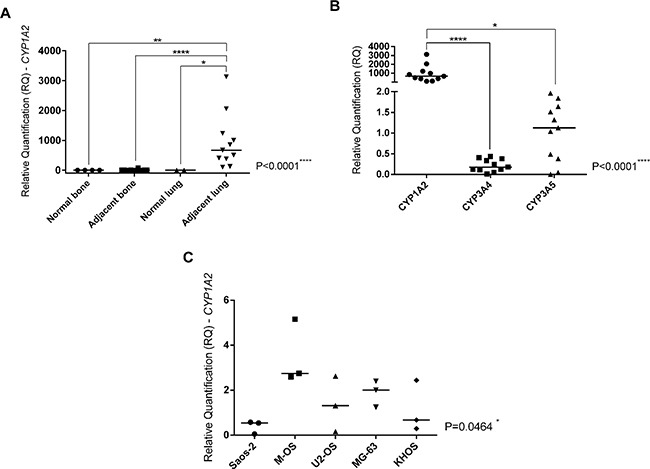
*CYP1A2* expression and its correlation with OS metastasis and metastatic tumor microenvironment **A**. Kruskal-Wallis Test (p<0.0001). Analyses between two groups were done by Mann-Whitney Test that confirmed *CYP1A2* overexpression in adjacent lung to OS metastasis in comparison with normal bone (control of the bone tissue) (p=0.0015), adjacent bone to primary OS (p<0.0001) and normal lung (control of the lung tissue) (p=0.0256). **B**. Friedman Test (p<0.0001). Dunn's multiple comparisons test confirmed *CYP1A2* overexpression in comparison with *CYP3A4* (p<0.0001) and *CYP3A5* (p=0.0168) in adjacent lung to OS metastasis. **C**. Kruskal-Wallis Test. *CYP1A2* overexpression in M-OS cell line, established from OS metastasis in the lung (p=0.0464). Statistical significance: *: p<0.05; **: p<0.01; ***: p<0.001; ****: p<0.0001.

*CYP1A2* gene expression and its association with pulmonary microenvironment in OS metastasis could be observed in Figure 1. The adjacent lung to pulmonary OS metastasis specimens presented *CYP1A2* overexpression compared to normal lung (p=0.0256), adjacent bone (p<0.0001) and normal bone (p=0.0015) specimens (Figure 1A). The *CYP1A2* gene overexpression observed in adjacent lung to OS metastasis specimens was exclusive for this *CYP* gene among the others investigated, as *CYP3A4* (p<0.0001) and *CYP3A5* (p=0.0168).

**Figure 2:**
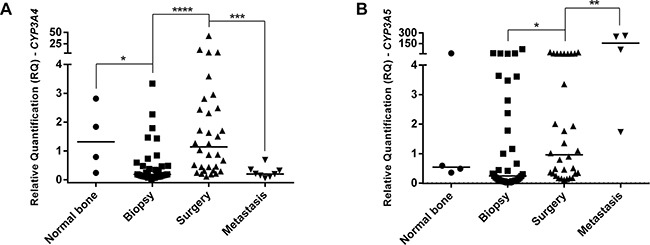
*CYP3A4* and *CYP3A5* genes expression in OS specimens and normal bone **A**. *CYP3A4* gene expression. Biopsy presented lower expression than normal bone specimens. Surgery specimens presented higher expression than biopsy. First metastasis from each patient presented lower expression than surgery and normal bone specimens, presenting similar expression to biopsy. **B**. *CYP3A5* gene expression. Surgery specimens presented overexpression than biopsy and lower expression than metastasis at diagnosis specimens. Statistical significance by Mann-Whitney and Wilcoxon Test: *: p<0.05; **: p<0.01; ***: p<0.001; ****: p<0.0001.

It was also observed a difference in *CYP3A4* and *CYP3A5* gene expression in analyzed specimens (Figure 2A and 2B, respectively). Biopsy specimens presented lower *CYP3A4* expression than normal bone specimens (p=0.0314). Surgery specimens presented *CYP3A4* overexpression than biopsy (p<0.0001) and first occurred metastasis specimens from each patient (p=0.0006). In *CYP3A5* gene expression analyses, surgery specimens presented higher expression than biopsy specimens (p=0.0157) and lower expression than metastasis at diagnosis specimens (p=0.0066).

**Figure 3:**
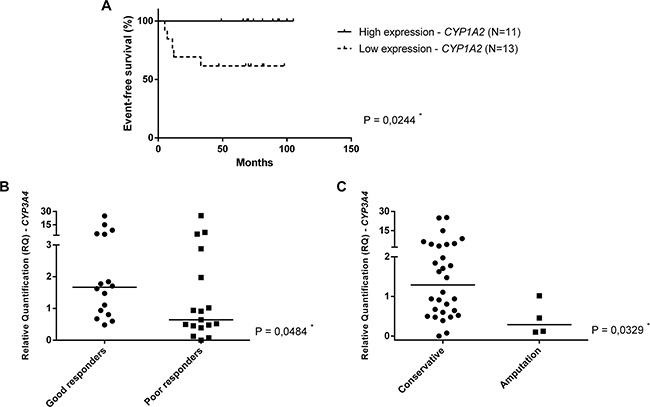
*CYPs* gene expression and outcome in OS **A**. Event-free survival of nonmetastatic at diagnosis patients according to high expression (more or equal than median value) and low expression (less than median value) of *CYP1A2* gene. The median value of *CYP1A2* relative quantification was 0.3797 in surgery specimens. **B**. Mann-Whitney Test. *CYP3A4* expression in adjacent bone specimens (post-chemotherapy), according tumor necrosis grade. **C**. Mann-Whitney Test. *CYP3A4* expression in adjacent bone specimens (post-chemotherapy), according to type of surgery.

Furthermore, *CYPs* gene expression showed to be associated with the outcome of OS patients (Figure 3). The survival curves of OS patients were analyzed according high expression (more or equal than median value) and low expression (less than median value) of *CYP* genes. In this analysis, we have separated two independent groups of patients: metastatic and nonmetastatic at diagnosis, since metastasis at diagnosis is an important prognostic factor in OS. When only nonmetastatic patients at diagnosis were analyzed, the *CYP1A2* overexpression in surgery specimens was associated with a better event-free survival (p=0.0244) (Figure 3A). Also, *CYP3A4* overexpression in adjacent bone specimens was associated with good response (tumor necrosis grade higher than 90% - grades III and IV) (p=0.0484) and conservative surgery (p=0.0329) (Figure 3B and 3C, respectively).

### Gene expression of *CYP1A2*, *CYP3A4* and *CYP3A5* in OS cell lines untreated and treated with chemotherapeutic drugs

**Figure 4:**
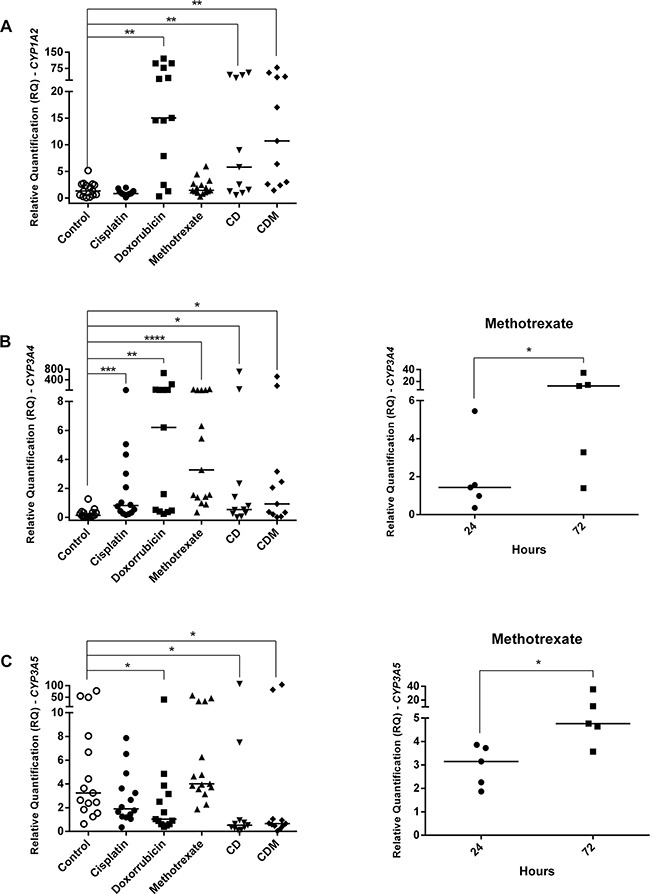
*CYPs* gene expression in OS cell lines treated with cisplatin, doxorubicin, methotrexate, cisplatin plus doxorubicin (CD), and cisplatin plus doxorubicin plus methotrexate (CDM) **A**. *CYP1A2* upregulation by doxorubicin, CD and CDM treatments. **B**. *CYP3A4* upregulation by cisplatin, doxorubicin, methotrexate, CD and CDM treatments, whereas upregulation by methotrexate was time-dependent. **C**. *CYP3A5* downregulation by doxorubicin, CD and CDM treatments, whereas methotrexate upregulated in a time-dependent manner. Statistical significance by Mann-Whitney and Wilcoxon: *: p<0.05; **: p<0.01; ***: p<0.001; ****: p<0.0001.

Although all OS cell lines have been treated with IC_50_, some samples did not show a good quality of RNA, probably due to drugs effects, thus they were excluded from analyses. The present investigation showed that the M-OS cell line untreated presented *CYP1A2* overexpression than other OS cell lines (p=0.0464) (Figure 1C). Analyses of *CYP* genes expression in OS cell lines untreated (control) and treated with cisplatin, doxorubicin, methotrexate, cisplatin plus doxorubicin (CD), cisplatin plus doxorubicin plus methotrexate (CDM) were showed in Figure 4. The doxorubicin, CD and CDM treatments upregulated the *CYP1A2* expression in OS cell lines (p=0.0034, p=0.0068 and p=0.0020, respectively) (Figure 4A). All the five treatments (cisplatin, doxorubicin, methotrexate, CD and CDM) upregulated the *CYP3A4* expression in OS cell lines (p=0.0004, p= 0.0024, p<0.0001, p=0.0472 and p=0.0362, respectively) (Figure 4B). The *CYP3A5* expression in OS cell lines was downregulated after treatments with doxorubicin, CD and CDM (p=0.0285, p=0.0463 and p=0.0108, respectively) (Figure 4C). Additionally, treatment with methotrexate showed an increased expression of *CYP3A4* and *CYP3A5* genes in a time-dependent manner (p=0.0085 and p=0.0239, respectively) (Figure 4B and 4C), in which 72 hours of treatment resulted in higher gene expression than 24 hours of treatment.

## DISCUSSION

The CYP enzymes are involved in the biosynthesis or catabolism of several endogenous compounds involved in a variety of important pathways [11]. Furthermore, CYP members can detoxify anticancer drugs used in OS treatment, such as doxorubicin and cisplatin [5]. Thus, CYP members could play a role in both tumorigenesis and treatment response [4]. To the best of our knowledge, this is the first study that investigated *CYP* gene expression in primary and metastatic OS, as well as in nonmalignant tissue surrounding the tumor, in order to investigate *CYP* role in priming tumor microenvironment and tumorigenesis in OS. Also, for the first time *CYP* gene expression was correlated with the outcome in OS and the modulation of its expression was investigated *in vitro* by the main chemotherapeutic drugs used in OS treatment.

The present study revealed that adjacent lung specimens presented *CYP1A2* overexpression, but not *CYP3A4* and *CYP3A5*, when compared to normal lung specimens. It was also observed that biopsy specimens presented lower *CYP3A4* gene expression than normal bone specimens. Surgery specimens, representing post-chemotherapy tumors, showed both *CYP3A4* and *CYP3A5* overexpression. Metastasis specimens presented lower *CYP3A4* expression, but higher *CYP3A5* expression, than surgery specimens. The current analysis showed that surgery specimens with *CYP1A2* overexpression are associated with a better event-free survival in nonmetastatic patients at diagnosis. Furthermore, adjacent bone specimens showed *CYP3A4* gene overexpression in patients that presented good response or were submitted to conservative surgery. *In vitro* assays showed that *CYP1A2* expression was upregulated by doxorubicin; *CYP3A4* expression was upregulated by cisplatin, doxorubicin and methotrexate; and *CYP3A5* expression was downregulated by doxorubicin and upregulated in a time-dependent manner by methotrexate.

The *CYP1A2* overexpression exclusively in the adjacent lung tissue showed that this CYP member might contribute to a favorable microenvironment in the lung during OS metastasis establishment. Interestingly, the M-OS cell line, established from pulmonary OS metastasis, also showed *CYP1A2* overexpression, which emphasizes a possible correlation between *CYP1A2* and pulmonary OS metastasis. Polymorphisms in the *CYP1A2* gene have already been correlated with cancer risk in liver, lung, stomach, pancreas, breast, endometrium and ovarian [13–19]. Several meta-analyses showed that the polymorphism 164A>C in *CYP1A2* gene can result in 2–3 fold increase in the CYP1A2 activity, which has been associated with increased cancer risk, primarily lung cancer in Caucasians [24–27]. Interestingly, 82% of the adjacent lung specimens analyzed in the present study were obtained from Caucasians.

The CYP1A2 enzyme could contribute to an ideal microenvironment in the lung through several ways. First, benzo[a]pyrene, a CYP1 enzyme substrate and a PAH present in organic material combustion, such as, diesel and food, has implicated in the induction of cell proliferation as well as tumors including OS [28]. Moreover, CYP1 metabolism of both xenobiotics and endobiotics leads to reactive oxygen species (ROS) formation, which can cause genotoxicity, and lead to inflammation, another source of ROS formation [29]. Furthermore, a regulatory feedback loop between *AHR* (aryl hydrocarbon receptor) and *CYP1* genes is known, thus AhR interacts with nuclear factor κb (NFκB), estrogen receptor 1 (ESR1) and retinoblastoma 1 (RB1), which triggers the transcription of genes involved in growth, cell cycle and apoptosis, leading to abnormal growth and tumor promotion [29]. For example, the AHR/RB1 complex, could cause RB1 hyperphosphorylation and cell cycle progression [30]. Also, it is already known a strong correlation between *RB1* mutations and OS tumorigenesis [31, 32], showing that AHR/CYP1 modulation could influence OS tumorigenesis and maintenance.

The low *CYP3A4* expression in the biopsy suggested its role in OS tumorigenesis. The CYP3A4 enzyme has the highest abundance in the human liver (~40%) [33] and is involved in the oxidative deactivation of testosterone [34, 35]. Testosterone plays a crucial role in bone metabolism and pubertal growth spurt [36], and it could be mediated by activation via Akt (serine/threonine kinase) and MAPK (mitogen-activated protein kinase) [37]. In this sense, *MAPK7* has already been studied in OS and might be a promising therapeutic target for this tumor, since modulates growth, proliferation and migration in OS [38].

As we observed increase of the *CYP3A4* expression in surgery specimens, we propose that it might have occurred due to chemotherapy treatment. However, metastasis specimens showed lower *CYP3A4* expression than surgery and normal bone specimens, demonstrating a similar pattern observed in biopsy specimens. On the other hand, the upregulation of *CYP3A5* expression not only occurred when surgery and biopsy specimens were compared, but also when metastasis specimens were compared with normal bone, biopsy and surgery specimens. Although the transcriptional regulation of *CYP3A4* and *CYP3A5* genes are done by the same receptor, PXR [39], the present study showed a similar *CYP3A4* expression in biopsy and metastasis specimens, which could be related to some resistance mechanism.

Our findings as to *CYP* overexpression and good outcome in OS patients are in agreement with other CYP studies of drug toxicity [29]. The correlation between *CYP1A2* overexpression and better event-free survival, probably is due to doxorubicin toxicity mediated by CYP1A2 enzyme against OS cancer cells, since CYP1A2 metabolizes this chemotherapy drug [40]. The association between *CYP3A4* overexpression and good response objected to what has been found by Dhaini et al., which showed that high CYP3A4/5 expression may predict metastasis and poor prognosis in OS [7]. The contrast could be explained by differences in analyzed samples. The present study showed an association with post-chemotherapy specimens, while Dhaini et al. investigated only pre-chemotherapy samples. Moreover, the present results regarding patients' specimens and *in vitro* assays, demonstrated that chemotherapy upregulates *CYP3A4*, which enzyme participates in cisplatin and doxorubicin metabolism [5].

*In vitro* assays showed that treatments with doxorubicin, CD or CDM upregulated *CYP1A2* expression in OS cell lines. In H9c2 cell lines, derived from heart, there was already reported a significant induction of *CYP1A2* expression by doxorubicin, as well as in breast cancer MCF7 cell line doxorubicin-resistant [40, 41]. These findings could be explained by AhR protein induction by doxorubicin, once *CYP1A2* gene is coordinately regulated at the transcriptional level by AhR protein [42]. Regarding *CYP3A4* expression, it was observed upregulation in OS cell lines by all five treatments. Moreover, cell lines treated with methotrexate showed *CYP3A4* upregulation time-dependent, whereas the treatment for 72 hours presented higher expression than 24 hours. This regulation could be mediated by PXR, which is the major responsible for *CYP3A4* gene transcription [10, 12] and has already known to be expressed in OS cell lines, such as HOS, MG-63, and Saos-2 [43].

Although the transcriptional regulation of *CYP3A4* and *CYP3A5* genes are performed by the same receptor, PXR [39], our findings *in vitro* showed that *CYP3A4* and *CYP3A5* genes were differently regulated by chemotherapy. Treatment with doxorubicin, CD and CDM in OS cell lines, different of *CYP3A4*, downregulated *CYP3A5* expression. However, it was observed *CYP3A5* overexpression in post-chemotherapy OS specimens. We suggest that methotrexate upregulated *CYP3A5* expression in patients' specimens, due to *in vitro* assays have shown *CYP3A5* overexpression in 72 hours of methotrexate treatment than in 24 hours. Therefore, a longer time of methotrexate treatment could increase even more *CYP3A5* expression. Thus, *CYP3A5* gene upregulation by methotrexate could prevail over downregulation by doxorubicin, since methotrexate is administrated in patients twice per cycle of chemotherapy and doxorubicin is administrated only once per cycle.

The CYP enzymes played controversial roles during time. In the pre-1968 era, CYP enzymes were thought to be beneficial because of detoxification, in the 1968–1998 era everyone was convinced that CYP enzymes were always detrimental, and since 1998 it seems more protective than destructive during environmental injury [29]. So, these controversial opinions probably were due to its great importance in several metabolism and molecular pathways. Thus, the present study showed that *CYPs* gene expression could play a role in different ways, depending if their influence was considered in tumorigenesis or treatment response. Due to that, *CYP* genes should be more explored in OS, a complex disease, to clarify these correlations.

In conclusion, the present study showed that adjacent lung to OS metastasis presented *CYP1A2* overexpression, indicating its role in priming a favorable microenvironment during the establishment of OS lung metastasis. Moreover, biopsy specimens presented lower *CYP3A4* expression than normal bone, which suggests a possible correlation between *CYP3A4* and OS tumorigenesis. The *CYP1A2* and *CYP3A4* overexpression post-chemotherapy were correlated with a better event free-survival and a better treatment response, respectively, probably due to the role of CYP1A2 and CYP3A4 enzymes in chemotherapy drugs metabolism. Additionally, in OS cell lines was verified that the own drugs used in OS treatment modulated *CYPs* gene expression. Therefore, the present study suggests that *CYP* genes play an important role in OS tumorigenesis, at primary and metastatic sites, as well in treatment response.

## MATERIALS AND METHODS

### Patients and specimens

We investigated 135 specimens from 37 patients with the diagnosis of OS admitted to treatment in the Pediatric Oncology Institute (IOP/GRAACC/UNIFESP), between 2006 and 2011, with an average of 16 years of age at diagnosis. Thus, the study included 37 biopsies (pre-chemotherapy), 37 OS resected in surgery (post-chemotherapy), 37 nonmalignant bone tissues surrounding OS resected in surgery (adjacent bone - post-chemotherapy), 13 lung metastases and 11 nonmalignant lung tissues surrounding OS metastases (adjacent lung). Lung metastasis and adjacent lung specimens were obtained from eight patients, in which four were metastatic patients at diagnosis. Four normal bone tissues were used as control, they were obtained from orthopedic surgeries of four healthy individuals that suffered a trauma and did not present either genetic disorders or bone diseases. Two different samples were used as control for the nonmalignant lung tissues surrounding metastases: one normal lung from a healthy individual without genetic disorders and one human lung total RNA from Thermo Fisher Scientific (Waltham, MA, USA). This study had the Research Ethics Committee approval from the Federal University of Sao Paulo (CEP UNIFESP 833694) and all patients agreed to participate by an informed consent. All patients were treated following the GLATO (Grupo Latino Americano de Tratamento de Osteossarcoma - Latin American Group of Osteosarcoma Treatment) protocol of 2006, which is based on high doses of cisplatin, doxorubicin and methotrexate. All clinical data are summarized in Table 1.

**Table 1:** Clinical features of OS patients

Clinical features	No. (%)
Metastasis at diagnosis	
Yes	6 (16)
No	31 (84)
**Location**	
Femur	22 (59)
Tibia	8 (22)
Humerus	3 (8)
Others	4 (11)
**Size**	
< 12 cm	26 (70)
≥ 12 cm	11 (30)
**Histology**	
Osteoblastic	18 (49)
Condroblastic	4 (11)
Fibroblastic	3 (8)
Mixed	7 (19)
Others	5 (13)
**Grade of tumor necrosis**	
< 90%	18 (49)
≥ 90%	17 (46)
Not identified	2 (5)
**Surgery**	
Conservative	32 (87)
Amputation	5 (13)
**Relapse**	
Yes	10 (27)
No	27 (73)
**Local of relapse**	
Lung	7 (19)
Bone	4 (11)
**Metastasis**	
Yes	11 (30)
No	26 (70)
**Local of metastasis**	
Lung	10 (27)
Bone	3 (8)
**Status**	
Alive	29 (78)
Dead	8 (22)

### Cell lines

The human OS cell lines Saos-2, KHOS, MG-63, and U2-OS were purchased from the American Type Culture Collection (Rockville, MD, USA). The human cell line M-OS was established in our laboratory from a OS lung metastasis at diagnosis [44]. All cell lines were cultured in Dulbecco's Modified Eagle Medium (DMEM) supplemented with 10% fetal bovine serum (FBS). All cells were cultured in a humidified incubator at 37°C and 5% CO_2_.

### Cytotoxicity assay and IC_50_

The half maximal inhibitory concentration (IC_50_) of each drug (cisplatin, doxorubicin and methotrexate) and their combinations were determined by the cytotoxicity assay. It was considered two combinations used during OS treatment: 1) cisplatin and doxorubicin (CD), combination used in the first chemotherapy of each cycle; 2) cisplatin, doxorubicin and methotrexate (CDM), considering together all drugs used in this treatment.

The cells were plated in a 96-wells plates with 100 μl/well and 5×10^3^ cells/well, except Saos-2 (1×10^4^ cells/well) and performed in triplicate for all cell lines. After overnight incubation to adhesion of cells, the medium was removed and drugs were added in serial concentrations (0.39–200 μg/ml). Untreated cells were used as control. After 24, 48, and 72h of incubation, the medium with drugs was removed and each well was washed with 200 μl of phosphate-buffered saline (PBS). Then, 100 μl of PrestoBlue® Cell Viability Reagent (Invitrogen, Waltham, MA, USA) dissolved in DMEM was added in each well. After 2h of incubation, all plates were analyzed on the ELISA reader M3 Spectra Max (Molecular Devices, Sunnyvale, CA, USA). The IC_50_ values were calculated for every treatment and time.

### qRT-PCR

All frozen tissues were submitted to RNA extraction using TRIzol® Reagent (Thermo Fisher Scientific, Waltham, MA, USA). All treated and untreated OS cell lines were submitted to RNA extraction using NucleoSpin Triprep® Kit (Macherey-Nagel, Duren, Germany). The cDNA was synthesized using SuperScript® Vilo™ Master Mix (Invitrogen, Waltham, MA, USA). The gene expression was measured by quantitative reverse transcription PCR (qRT-PCR) using SYBR® Green PCR Master Mix (Applied Biosystems™, Waltham, MA, USA). The *ACTB* and *GAPDH* genes were used as endogenous controls. Normal bone and normal lung were used as calibrators. All primers were designed using online tools (Clustal and NCBI primer express) and their sequences were: *ACTB* forward 5′-AAGGCCAACCGCGAGAAG-3′, reverse 5′-ACAGCCTGGATAGCAACGTACA-3′; *GAPDH* forward 5′-ACAACTTTGGTATCGTGGAAGGA-3′, reverse 5′-TCTTCTGGGTGGCAGTGATG-3′; *CYP1A2* forward 5′-GCCTGAGATACAGAGGAAGATCCA-3′, reverse 5′-CGCTCCCTGCCAATCACA-3′; *CYP3A4* forward 5′-TATGCTCTTCACCGTGACCCAA-3′, reverse 5′-CCTTGTTCTTCTTGCTGAAT-3′; and *CYP3A5* forward 5′-CCTCTGCCTTTGTTGGGAAATG-3′, reverse 5′- GCACTCTGTGTCAAATTTCCAGAG-3′. The relative quantification (RQ) was calculated by the 2^−ΔΔCt^ method.

### Statistical analyses

Data analyses was performed using the GraphPad Prism version 6.0 for Windows (GraphPad Software, San Diego, CA, USA). IC_50_ values were calculated by the software. The gene expression, which is represented by the relative quantification, was compared using nonparametric tests: Wilcoxon, Mann-Whitney, Friedman, Kruskal-Wallis and Dunn's multiple comparisons. The overall and event-free survivals were calculated by the Kaplan-Meier method and the survival curves were compared by the log-rank test. Statistical significance was taken when P<0.05.
